# Chi3l3: a potential key orchestrator of eosinophil recruitment in meningitis induced by *Angiostrongylus cantonensis*

**DOI:** 10.1186/s12974-018-1071-2

**Published:** 2018-02-02

**Authors:** Shuo Wan, Xiaoqiang Sun, Feng Wu, Zilong Yu, Lifu Wang, Datao Lin, Zhengyu Li, Zhongdao Wu, Xi Sun

**Affiliations:** 1 Department of Parasitology of Zhongshan School of Medicine, Sun Yat-sen University, No.74 Zhongshan Road.2, Guangzhou, Guangdong 510080 China; 2 Key Laboratory of Tropical Disease Control (SYSU), Ministry of Education, Guangzhou, Guangdong 510080 China; 3 Provincial Engineering Technology Research Center for Biological Vector Control, Guangzhou, Guangdong 510080 China; 4 Department of Clinical Laboratory, the Sixth Affiliated Hospital, Sun Yat-Sen University, Guangzhou, Guangdong 510080 China; 5 Institute of Human Disease Genomics, Zhongshan School of Medicine, Sun Yat-Sen University, Guangzhou, Guangdong 510080 China; 6 Department of neurology, The Second Affiliated Hospital of Nanchang University, Nanchang, Jiangxi 330000 China

**Keywords:** Brain, Eosinophilic infiltration, Macrophage, Polarization, Soluble antigens of *A. cantonensis* larvae (L4) (sAg), Chi3l3-IL-13

## Abstract

**Background:**

*Angiostrongylus cantonensis*, an important foodborne parasite, can induce serious eosinophilic meningitis in non-permissive hosts, such as mouse and human. However, the characteristics and mechanisms of the infection are still poorly understood. This study sought to determine the key molecules and its underlying mechanism in inducing brain eosinophilic infiltration caused by *Angiostrongylus cantonensis*.

**Methods:**

Mathematical models were established for prediction of significantly changing genes and the functional associated protein with RNA-seq data in *Angiostrongylus cantonensis* infection. The expression level of Chi3l3, the predicted key molecule, was verified using Western blotting and real-time quantitative PCR. Critical cell source of Chi3l3 and its relationship with eosinophils were identified with flow cytometry, immunohistochemistry, and further verified by macrophage depletion using liposomal clodronate. The role of soluble antigens of *Angiostrongylus cantonensis* in eosinophilic response was identified with mice airway allergy model by intranasal administration of *Alternaria alternate*. The relationship between Chi3l3 and IL-13 was identified with flow cytometry, Western blotting, and Seahorse Bioscience extracellular flux analyzer.

**Results:**

We analyzed the skewed cytokine pattern in brains of *Angiostrongylus cantonensis*-infected mice and found Chi3l3 to be an important molecule, which increased sharply during the infection. The percentage of inflammatory macrophages, the main source of Chi3l3, also increased, in line with eosinophils percentage in the brain. Network analysis and mathematical modeling predirect a functional association between Chi3l3 and IL-13. Further experiments verified that the soluble antigen of *Angiostrongylus cantonensis* induce brain eosinophilic meningitis via aggravating a positive feedback loop between IL-13 and Chi3l3.

**Conclusions:**

We present evidences in favor of a key role for macrophave-derived Chi3l3 molecule in the infection of *Angiostrongylus cantonensis*, which aggravates eosinophilic meningitis induced by *Angiostrongylus cantonensis* via a IL-13-mediated positive feedback loop. These reported results constitute a starting point for future research of angiostrongyliasis pathogenesis and imply that targeting chitinases and chitinase-like-proteins may be clinically beneficial in *Angiostrongylus cantonensis*-induced eosinophilic meningitis.

**Electronic supplementary material:**

The online version of this article (10.1186/s12974-018-1071-2) contains supplementary material, which is available to authorized users.

## Background

*Angiostrongylus cantonensis* (*A. cantonensis*, AC), a rat lung nematode, is a serious foodborne parasite. It occurs in Asia, the Pacific islands, Australia, and the Caribbean islands [1]. Humans and mice, non-permissive hosts, become infected by eating raw intermediate hosts, including *Pomacea canaliculate* and *Ampullaria crossean* [2]. In recent years, due to the wide spread of snails and slugs, the disease is no longer restricted to certain areas [3, 4]. AC has become a major threat to both human beings and wildlife species globally. Two nine-banded armadillos and one Virginia opossum were reported to be infected with AC in the southeastern USA [5]. According to a review published in 2008, nearly 3000 cases of human angiostronglyiasis had been documented worldwide [6]; however, this number has risen rapidly. In a prospective descriptive study conducted from June 2008 to January 2014 in a Vietnamese hospital, AC was an important cause of eosinophilic meningitis, accounting for 67.3% (37/55) of cases [7].

Most individuals develop eosinophilic meningitis when infected with AC, and common clinical symptoms include headache, neck stiffness, paresthesia, vomiting, and nausea. Furthermore, if the worms move to the eyes, ocular angiostrongyliasis may occur, causing visual disturbances. Additionally, surgery must be performed to remove the worms from the eyes of patients with ocular angiostrongyliasis [8]. Thus far, treatment of angiostrongyliasis is still limited. Traditional anthelmintic drugs, such as albendazole and mebendazole, are not recommended for angiostrongyliasis treatment, as they may exacerbate neurological symptoms [9].

Although AC may produce severe neurological disease, little is known about its underlying pathogenic mechanisms. It is widely assumed that type 2 immunity, a major protective mechanism against helminth infection, such as filarial nematode *Brugia malayi* [10], *Nippostrongylus brasiliensis* [11], *Trichinella spiralis* [12], *Schistosoma japonicum* [13], and *Heligmosomoides polygyrus* [14], is involved in the process of helminth infection. And IL-5 [15, 16], IL-13 [17], and Eotaxin [18, 19] are currently regarded as the common characteristics in eosinophilic infiltration-related diseases. Unexpectedly, *Mesocestoides corti* infection in IL-5 knockout mice resulted in normal blood and tissue eosinophil levels [20]. In addition, knockout of Eotaxin [21] partially reduced antigen-induced tissue eosinophilia. CCR3 [22, 23], as the receptor of Eotaxin, also played an important role in eosinophil migration to injured tissues, suggesting that other factors may participate in eosinophil infiltration as well.

Chi3l3, an unconventional eosinophil-related protein [24], is highly expressed in Th2 type immune responses caused by helminth infections and allergic diseases. Here, we identified burst expression of Chi3l3 as a useful discriminative marker between the non-permissive host and permissive host, as well as a key player during AC infection in the non-permissive mouse model host. We also investigated its biological function during AC-induced eosinophilic meningitis.

## Methods

### Preparation of soluble antigens of AC (sAg) and bone marrow-derived macrophage (BMDM) cells

Soluble antigens of the 4th stage larvae of AC (AC L4) were collected from Sprague-Dawley rat brains at 21 dpi as previously described [25]. Bone marrow cells were isolated as described previously [26] and cultured with M-CSF (20 ng/mL) in complete medium [DMEM High Glucose, 100 U/ml of penicillin-streptomycin and 10% heat-inactivated (56 °C, 30 min) FBS] for 7 days. The following reagents were used in this study: Recombinant Murine M-CSF (315-02; Peprotech), Recombinant Mouse Chi3l3 (2446-CH-050, R&D Systems), and Recombinant Mouse IL-13 (413-ML-025/CF, R&D Systems).

### Establishment of airway allergy and AC infection models

For establishment of the airway allergy model, female C57 mice were chosen, and *Alternaria alternata* (Greerlabs, Lenoir, NC, 100 g/mouse in 50 μL) or PBS (50 μL) was administered intranasally on four consecutive days [27]. Four hours before the daily *Alternaria alternata* administration, 50 μg soluble rat-derived antigens of AC larvae (L4) or an equal volume of PBS treatment was administered via nasal drip. On the day after the last intranasal stimulation, mice were euthanized and BAL was harvested. Then, the cells derived from BAL were stained by Siglec F PE (BD), CD11c PE-Cyanine5 (eBioscience), or CD45 APC-cy7 (Biolegend) and analyzed by flow cytometry [28]. For the AC infection model, each Sprague-Dawley rat and BALB/c mouse were orally infected with 100 and 30 AC larvae (L3), respectively. AC larvae (L3) were collected from the tissue of infected *Biomphalaria glabrata* as previously described [29]. The mice and Sprague-Dawley rats were euthanized on days 7, 14, 21, and 28 after infection with AC.

### Histological examination and immunohistochemistry

Brain and lung hematoxylin and eosin staining and immunofluorescence were performed on 5-mm-thick, 4% paraformaldehyde-fixed, paraffin-embedded slices. Antibodies against mouse Ym1 (60130, StemCell Technologies), CD11b (MAB1124, R&D Systems), and Iba1 (NCNP24, Wako) were used as primary antibodies, with the corresponding secondary antibodies labeled with FITC or Alexa Fluor® 594. In addition, nuclei of cells were displayed by DAPI staining. All tissue slices were examined with laser-scanning confocal microscopy (Zeiss LSM780; Germany).

### Flow cytometry analysis

Flow cytometry analysis was performed on a CytoFLEX S flow cytometer (Beckman Coulter).

For blood and brain macrophage and eosinophil assessment, PBMCs and BMNCs were isolated as previously described [25] and then incubated with Siglec F PE (BD), CD11c PE-Cyanine5 (eBioscience), or CD11b APC-cy7 (eBioscience) at 4 °C for 30 min.

To determine the cell sources of Chi3l3, BMNCs were stained with CD11b PE (eBioscience), F4/80 PE-Cyanine5 (eBioscience), CD45 PE-eFluor 610 (eBioscience), Ly-6C PerCP/Cy5.5 (Biolegend), CX3CR1 Alexa Fluor 488 (eBioscience), or CCR2 Phycoerythrin (R&D) at 4 °C for 30 min.

For measurement of IL-5 and IL-13 levels, spleen cells [30] from normal and AC-infected mice were incubated with CD3 APC (eBioscience), CD4 FITC (eBioscience), and IL-13 PE cy7 (eBioscience) at 4 °C for 30 min and analyzed by flow cytometry.

To determine the possible effects of Chi3l3 and sAg on IL-13 production, spleen cells isolated from normal and AC-infected mice were cultured in 24-well cell culture plates for 72 h, followed by a 12-h incubation with Brefeldin A and 6-h stimulation with PMA and ionomycin. Then, the cells were incubated with CD3 APC (eBioscience), CD4 FITC (eBioscience), or IL-13 PE cy7 (eBioscience) at 4 °C for 30 min and analyzed by flow cytometry.

### Cell metabolism measurement

OCR (Oxygen Consumption Rate) and ECAR (Extracelluar Acidification Rate), as indicators of cellular oxidative phosphorylation and glycolysis, respectively, were monitored consecutively with a Seahorse Bioscience extracellular flux analyzer (XF24, Seahorse Bioscience) as described previously [31]. Approximately 15,000–25,000 cells were seeded in 24-well plates cultured with 500 μL complete medium and incubated for 24 h in a 37 °C incubator. Then, cells were immersed in 500 μL specified medium following two wash steps with specified medium and incubated in an incubator without CO_2_ for 1 h before the measurements.

The basal levels of OCR were recorded thrice as were the OCR levels after sequential addition of 1 μM oligomycin, 1.0 μM FCCP, and 0.5 μM rotenone + 0.5 μM antimycin A. Similarly, ECAR was measured under basal conditions and after the injection of the following drugs: 10 mM glucose, 1.0 μM oligomycin, and 50 mM 2-deoxyglucose (2-DG).

### Protein and mRNA analysis

Mouse and rat brains were dissected into the cerebrum and cerebellum and stored in TRIzol at − 80 °C. Total RNA was extracted using TRIzol (Life Technologies) reagent and reverse-transcribed to cDNA using a RevertAid™ FirstStrand cDNA Synthesis Kit (Thermo Fisher Scientific, USA) according to the manufacturer’s protocol. Specific gene expression was quantified with SYBR® Premix Ex Taq™ (Tli RNaseH Plus) (RR420A) using the Roche LightCycler® 480 real-time PCR platform.

The following amplification primers (Sangon Biotech) were used (5′ to 3′): mouse Chi3l3 (sense, CTGAATGAAGGAGCCACTGA; antisense, AGCCACTGAGCCTTCAACTT), mouse β-actin (sense, GGCATCCTGACCCTGAAGTA; antisense, CTCTCAGCTGTGGTGGTGAA), rat Chi3l3 (sense, AGTACCCTATGCCGTTCAGG; antisense, CAGACCATTGCACCTCCTAA), rat B2m (sense, GTCACCTGGGACCGAGAC; antisense, GAAGATGGTGTGCTCATTGC), and rat Hprt1 (sense, CTGTCATGTCGACCCTCAGT; antisense, GTCCATAATCAGTCCATGAGGA).

The cerebrums and cerebellums of mice and rats were lysed using RIPA Lysis Buffer (Strong) (Cwbiotech), followed by SDS-PAGE. After transfer to nitrocellulose membranes (GE Healthcare) using the Semi-Dry Transfer with Trans-Blot® SD Semi-Dry Transfer Cell (Bio-Rad), target proteins were verified with the corresponding antibodies. The following primary antibodies were used: mouse JMJD3 (ab85392, Abcam), CREB1 (A11989, ABclonal), CEBPB (A0711, ABclonal), KLF4 (A11853, ABclonal), Y641 phosphorylated-STAT6 (ab54461, Abcam), STAT6 (5397, Cell Signaling Technology), Chi3l3 (#60130, STEMCELL Technology), PPARγ (81B8, Cell Signaling Technology), and β-actin (12262, Cell Signaling Technology).

### RNA-seq data collection and processing

Our group generated RNA-seq data of AC infection in mouse brain tissue [32]. The raw data were processed using a standard pipeline as follows: we first filtered the low-quality tags and trimmed adaptors. Next, we applied TopHat to map the clean reads to the *Mus musculus* 9 genome; we then used cufflinks and Cuffdiff to calculate the expression levels of transcripts and to analyze the differential expression, respectively.

### An algorithm for selecting marker genes from dynamic gene expression data

#### Selection of TCGs (significantly changing genes)

The RNA-seq data were measured on the 2nd, 7th, 14th, and 21st day post-infection. As gene expression temporally changed over time, we selected significantly changing genes by calculating the fold change of each gene between any two time points. For a given gene *G*_*i*_ at time points *t*_*j*_ (*j* = 1, 2, …, 5), if the following criteria were satisfied, then it was defined as a significant TCG:

- $$ \underset{k}{\max}\left({G}_i\left({t}_k\right)\right)\ge \theta $$;
- *G*_*i*_(*t*_*j*_)/*G*_*i*_(*t*_*k*_) ≥ *δ*_1_ or *G*_*i*_(*t*_*j*_)/*G*_*i*_(*t*_*k*_) ≤ 1/*δ*_1_.

That is, a given gene was defined as a TCG if the maximal expression level of this gene is greater than *θ* and the fold change of its expression level between two time points is greater than *δ*_1_. In this study, *θ* was set to 10, and *δ*_1_ was set to 5.

#### Clustering of expression patterns of TCGs

We performed an unsupervised hierarchical clustering of the expression levels of TCGs using the R package “hclust.” Then, the cluster tree was divided into six groups, and the gene expression pattern of each cluster was plotted.

#### Ranking of significantly changing genes

We ranked the significantly changing genes in the selected clusters (for example, sustained increasing or decreasing clusters) based on their changing rates at each time point. The changing rate of gene expression was defined as follows:

1 $$ {R}_i\left({t}_k\right)=\frac{G_i\left({t}_{k+1}\right)-{G}_i\left({t}_k\right)}{t_{k+1}-{t}_k} $$

where *G*_*i*_(*t*_*k*_) is the normalized expression level of gene *i* at time point *t*_*k*_. Then, the genes were ranked according to the maximal absolute value of their changing rates at all time points.

### Functional protein association networks and functional enrichment analysis

The functional protein association network for the genes in each cluster was constructed using the STRING database (https://string-db.org/). In this study, a medium confidence level (0.4) was selected to construct the network. We investigated the enriched functions of genes in the constructed network by performing functional enrichment using the DAVID webserver (https://david.ncifcrf.gov/). The adjusted *P* values were examined to select the significantly enriched biological processes.

### Mathematical model for Chi3l3-IL-13 positive feedback

We built a mathematical model to describe the dynamics of expression levels of Chi3l3 and IL-13 using ODEs as follows:

2 $$ \frac{d\left[ Chi3l3\right]}{dt}=a+\frac{V_1\cdot {\left[ IL13\right]}^{n_1}}{{K_1}^{n_1}+{\left[ IL13\right]}^{n_1}}-{d}_1\cdot \left[ Chi3l3\right] $$

3 $$ \frac{d\left[ IL13\right]}{dt}=b+\frac{V_2\cdot {\left[ Chi3l3\right]}^{n_2}}{{K_2}^{n_2}+{\left[ Chi3l3\right]}^{n_2}}-{d}_2\cdot \left[ IL13\right] $$

where [*Chi*3*l*3] and [*IL*13] represent the normalized expression levels of Chi3l3 and IL-13, respectively. *a* and *b* are the basic transcript rates of Chi3l3 and IL-13. *V*_1_ is the maximal activation rates of Chi3l3 promoted by IL-13, and *V*_*2*_ is that of IL-13 promoted by Chi3l3. *K*_1_ and *K*_2_ are the Michealis constants. *n*_1_ and *n*_2_ are Hill coefficients. *d*_1_ and *d*_2_ are degradation rates of Chi3l3 and IL-13, respectively.

The above ODEs were numerically solved using the 4th order Runge-Kutta method. The parameters in the above model was estimated using the non-linear least square method by fitting the model simulation to the experimental data. The experimental data were re-sampled using the 3rd order Hermite polynomial interpolation scheme. The genetic algorithm [33, 34] was employed to optimize the objective function. The estimated parameter values are as follows: *V*_1_ = 0.2588, *V*_2_ = 0.9686, *K*_1_ = 0.6980, *K*_2_ = 0.5216, *n*_1_ = 2, *n*_1_ = 1, *d*_1_ = 0.1216, *d*_2_ = 0.4824, *a* = 0.0039, and *b* = 0.0235. The programming was performed in MATLAB R2007b (MathWorks, USA). The bifurcation analysis was performed using Oscill8 (http://oscill8.sourceforge.net).

## Results

### Chi3l3 in mouse brains increased sharply during AC*-*induced eosinophilic meningitis

**Fig. 1:**
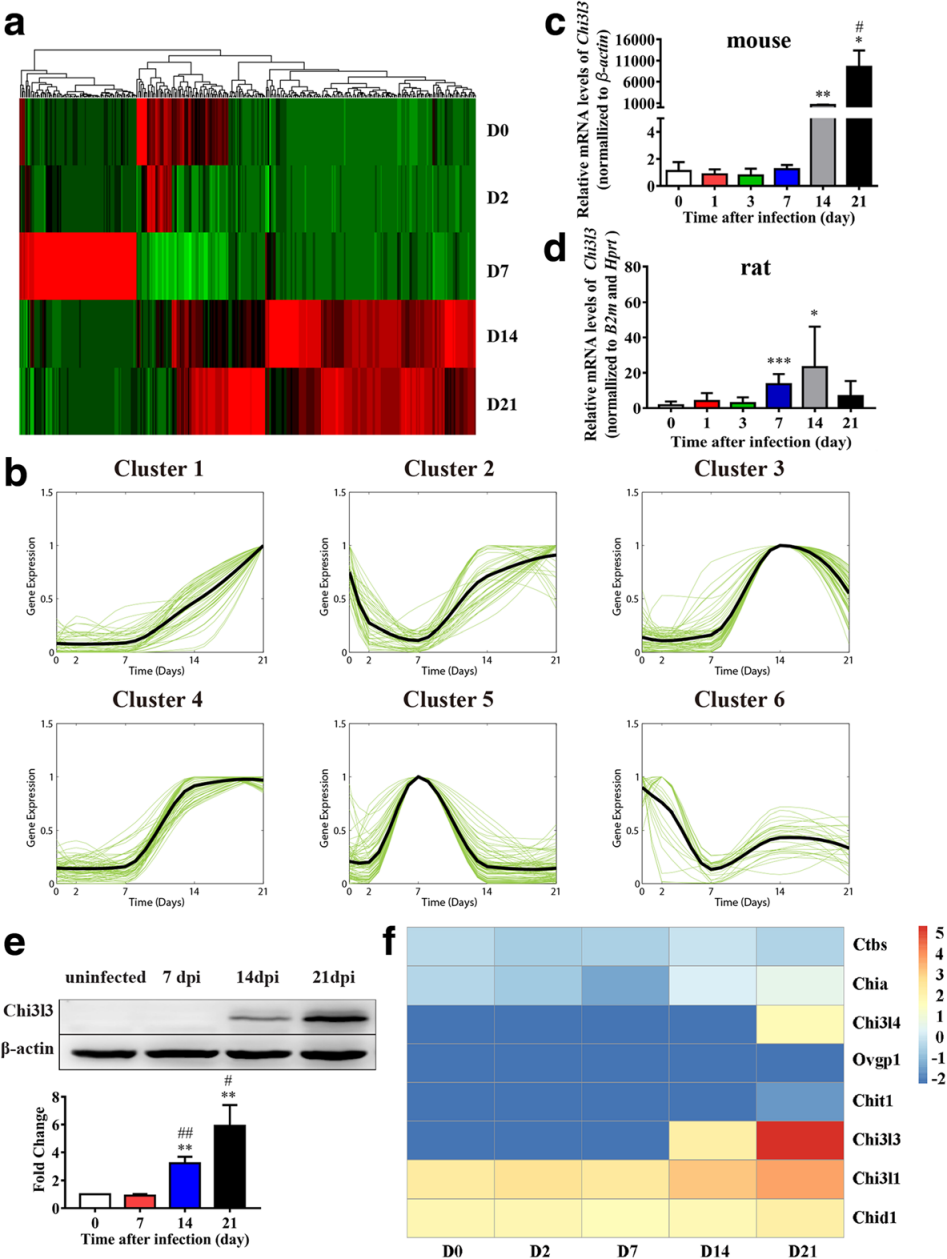
Elevation of Chi3l3 expression in the brain is an important characteristic in non-permissive host mice but not in permissive host rats during AC infection. **a** Expression profile of significant TCGs after AC infection. The horizontal axis represents genes, and vertical coordinates represents time points. D0 is the normal control group. D2, D7, D14, and D21 represent 2, 7, 14, and 21 dpi, respectively. **b** Expression patterns of TCGs. The *x*-axis represents time (with the unit as day), and the *y*-axis represents the normalized gene expression levels. **c** The mRNA levels of Chi3l3 in brains of mice infected with AC at 0, 1, 3, 7, 14, and 21 dpi. Mouse β-actin mRNA was used as an internal control. ^*^P < 0.05. infected groups vs control group; ^##^*P* < 0.01, 14 dpi group vs 21 dpi group. **d** The mRNA levels of Chi3l3 in brains of rats infected with AC at 0, 1, 3, 7, 14, and 21 dpi. Rat B2m and Hprt1 mRNA levels were used as internal controls. *P < 0.05, **P < 0.01, infected groups vs control group. **e** The protein levels of Chi3l3 in brains of mice infected with AC at 0, 7, 14, 21, and 28 dpi. β-Actin was used as an internal control. ^*^P < 0.05, ^**^P < 0.01, ^***^P < 0.001, infected groups vs uninfected groups in the corresponding part. **f** A gene set enrichment analysis of transcriptome data was performed by comparing the mRNA levels of CLPs and chitinases in brains of mice infected with AC at 0, 2, 7, 14, and 21 dpi. Data information: In **c**–**e**, data are presented as mean ± SD. (Student’s *t* test)

AC induced serious eosinophilic meningitis or meningoencephalitis when mice were infected with the third-stage larvae (*L3*) of AC. To elucidate the underlying mechanism of eosinophil infiltration in the brain, we analyzed the dynamic gene expression profiles of mouse brains using RNA-seq data [32]. We designed an algorithm for selecting significant temporally changing genes (TCGs), and 336 genes were identified as TCGs. The expression profiles of these TCGs are shown in a heatmap (Fig. 1a). TCGs were then clustered into six groups (Fig. 1b); each group had a different dynamic expression pattern. The cluster 1 genes showed a sustained increasing expression pattern after AC infection, indicating possible important functions. Therefore, we then analyzed the dynamic properties of cluster 1 genes by calculating their changing rates as defined in Eq. (1) in the “Methods” section. The maximal values of the changing rates of these genes at all time points were calculated and ranked from the largest to the smallest. We confirmed LOC547349 and Chi3l3 as the top two genes (Table 1), and Chi3l3 showed higher expression than LOC547349 (Additional file 1: Figure S1). Therefore, we hypothesized that Chi3l3 was an important molecule in AC infection. We further investigated the differences in Chi3l3 expression between non-permissive hosts (mice) and permissive hosts (rats) infected with AC and were surprised to find that the mRNA and protein levels of Chi3l3 in the brain varied greatly between mice and rats during infection (Fig. 1c, d). Mouse brain Chi3l3 mRNA levels increased from 1000 at 14 dpi to 10,000 at 21 dpi (Fig. 1d), and the protein level experienced a fold increase from 14 dpi to 21 dpi (Fig. 1e). Notably, AC infection time-dependently upregulated chitinases and chitinase-like proteins (CLPs) at 0, 2, 7, 14, and 21 dpi, with Chi3l3 being the most significant one (Fig. 1f), in accordance with our gene microarray data [35].

**Table 1:** Top 10 genes ranked according to the maximal changing rate of genes in cluster 1 of gene expression patterns

Gene symbols	Maximal changing rate	Maximal expression level
LOC547349	0.139800	22.7645
Chi3l3	0.136786	210.624
Aqp1	0.126326	12.3571
Apoc4	0.119975	12.3525
Mmp3	0.11429	10.435
Retnla	0.113539	1140.52
Ccl4	0.101572	34.1275
Ch25h	0.097177	15.4714
Serpina3h	0.096369	20.2997
S100a8	0.095943	127.981

Collectively, these results indicated that a sharp increase of Chi3l3 was observed in non-permissive host mice but not in permissive host rats during AC infection, and thus, Chi3l3 is likely to be an important element closely correlated with eosinophilic meningitis [24, 25, 35].

### Eosinophil percentage is coordinated with Chi3l3 derived from inflammatory macrophages in brains of AC-infected mice

**Fig. 2:**
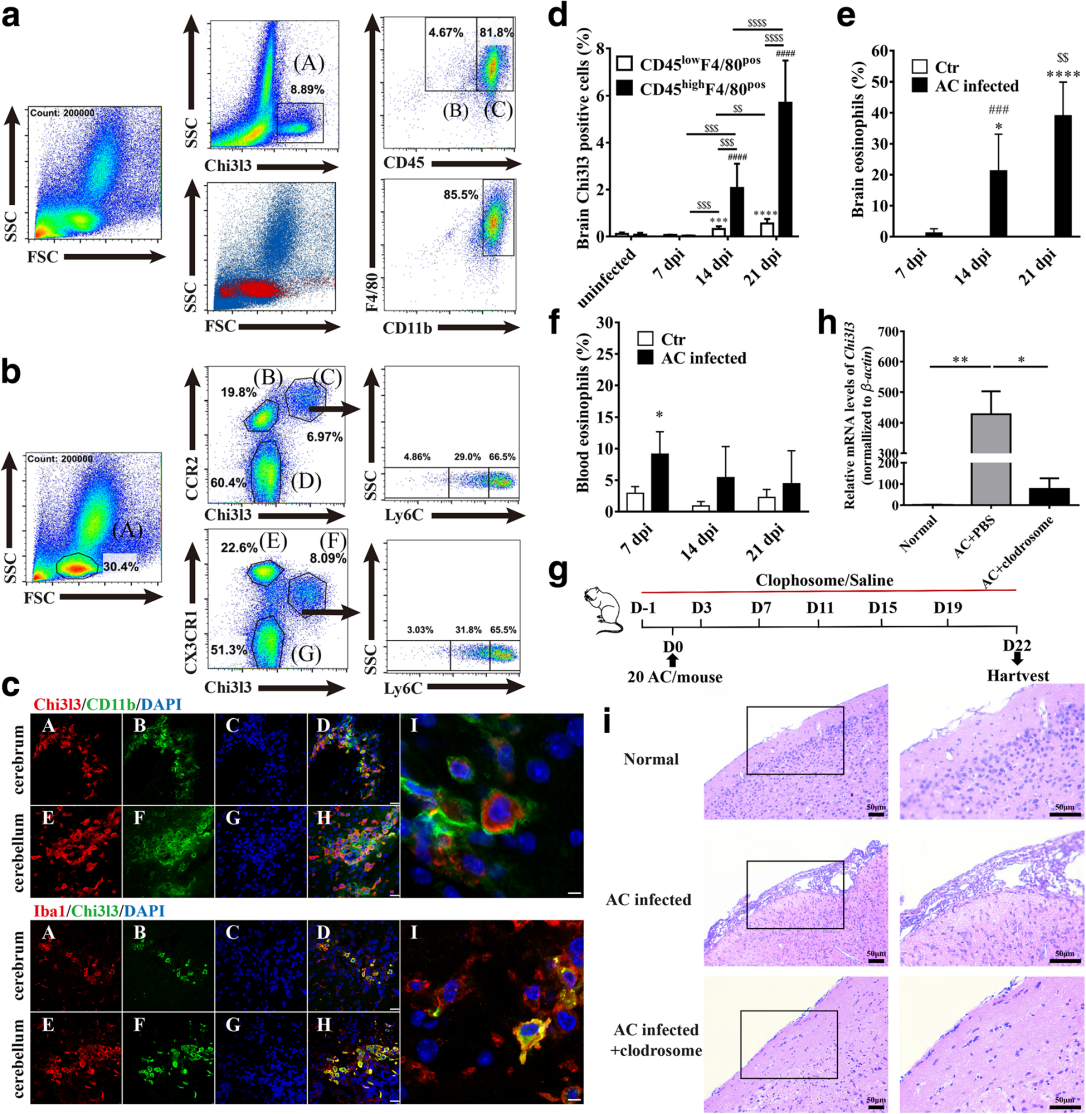
Eosinophil percentage coordinates with Chi3l3 derived from inflammatory macrophages in brains of AC-infected mice. **a** Chi3l3^+^ cells were observed in a gate of SSC and Chi3l3^+^ (A), within the gate (A). CD45^hi^F4/80^+^ and F4/80^+^CD11b^+^ populations are shown in (C) and (D), respectively. **b** Chi3l3^+^ cells were characterized by CCR2^+^ gate (C) or CX3CR1^+^ gate (F) separately. **c** (A–D) The co-localization of Chi3l3 or Iba1 (A), CD11b or Chi3l3 (B), and DAPI (C) in cerebrum of AC-infected mice. (D) is the merge of (A), (B), and (C). (E–H) Chi3l3 or Iba1 (E), CD11b or Chi3l3 (F), and DAPI (G) in the cerebellum of AC-infected mice. (H) is the merge of (E), (F), and (G). Scale bars indicate 20 μm. Scale bar in (I) indicates 5 μm. **d** The percentages of Chi3l3^+^CD45^lo^F4/80^+^cells in BMNCs of mice at 7, 14, and 21 dpi are presented. ^***^P < 0.001, ^****^P < 0.0001, AC-infected groups vs control group. Chi3l3^+^CD45^hi^F4/80^pos^ cells in BMNCs of mice at 7, 14, and 21 dpi are presented. ^####^*P* < 0.0001, AC-infected groups vs control group. ^$$^*P* < 0.01, ^$$$^*P* < 0.001, ^$$$$^*P* < 0.0001. **e** The percentages of eosinophils in BMNCs of mice infected with AC at 7, 14, and 21 dpi. There were 5–10 mice per group. Ctrl indicates the concurrent control. *P < 0.05, ****P < 0.0001, AC-infected groups vs control group. ^###^*P* < 0.001, AC-infected 14 dpi group vs AC-infected 7 dpi group. ^$$^*P* < 0.01, AC-infected 21 dpi group vs AC-infected 14 dpi group. **f** The percentages of eosinophils in peripheral blood mononuclear cells (PBMCs) of mice infected with AC at 7, 14, and 21 dpi; 5–10 mice per group. Ctrl indicates the concurrent control. *P < 0.05, AC-infected groups vs control group. **g** Clophosome and saline were injected into BALB/c mice intravenously 1 day before of AC infection, followed by five intravenous challenges every 4 days. At 22 dpi, the brain samples were collected. **h** Brain Chi3l3 mRNA levels (**g**). **i** Histopathological changes of the brain (**g**) (scale bar, 50 μm). Data information: In (**d-h**), data are presented as mean ± SD. (Student’s *t* test)

Excess Chi3l3 in the brain prompted us to investigate the possible cell source of this molecule. Flow cytometric analysis revealed that approximately 8% of brain mononuclear cells (BMNCs) of mice infected with AC at 21 dpi were Chi3l3-positive (Chi3l3^+^) cells. Further analysis showed that the majority (81.8%) of Chi3l3^+^ cells possess a CD45^hi^F4/80^+^CD11b^+^ phenotype (Fig. 2a). Monocytes/macrophages are currently divided into two classes, “inflammatory” type (CCR2^+^Ly-6C^hi^CD62L^+^CX3CR1^lo^) and “resident” type (CCR2^−^Ly-6C^lo^CD62L^−^CX3CR1^hi^) cells [36]. The above observation suggested that bone marrow-derived myeloid cells may be recruited to the inflamed CNS during AC infection, similar to other brain inflammatory diseases, such as multiple sclerosis and experimental autoimmune encephalomyelitis [37]. To test this hypothesis, we assessed CCR2, Ly-6C, and CX3CR1 as previously described [36]. Chi3l3^+^ BMNCs are primarily characterized by Ly-6C^+^CCR2^+^CX3CR1^lo^ and a low side scatter profile (Fig. 2b), which indicate an “inflammatory” subset of monocytes/macrophages, not a “resident” subset [36]. In addition, kinetics of CD45^+^ F4/80^+^ cell infiltration in the brain was evaluated; Chi3l3^+^ cells were first observed at 14 dpi, and the cell percentage increased sharply with eosinophilic meningitis progression (Fig. 2e), accompanied by enhanced expansion of CD45^hi^F4/80^+^ cells compared to CD45^lo^F4/80^+^ cells (Fig. 2d). Further evaluation of the macrophage-specific markers Iba1 [38] and CD11b by immunofluorescence revealed that infiltrating cells were differentiated mature macrophages after AC infection (Fig. 2c).

Intriguingly, we found that the eosinophil percentage synchronized with Chi3l3 and IL-13 levels in brains of AC-infected mice, with a percentage of approximately 2% at 7 dpi. The eosinophil percentage rose sharply over time, exceeded 20% at 14 dpi, and finally reached 40% at 21 dpi (Fig. 2e). In contrast to the CNS symptoms of AC*-*infected mice, we did not observe obvious changes in the eosinophil percentage in the blood of AC-infected mice at 14 dpi and 21 dpi compared with the uninfected healthy mouse group, although there was a slight increase in eosinophil percentage at 7 dpi (*P* < 0.05) (Fig. 2f).

To define the potential relationship between macrophage infiltration and eosinophilic meningitis in AC infection, we administered liposomal clodronate [39] prior to and during the infection to effectively deplete macrophages (Fig. 2g). As expected, treatment with liposomal clodronate (Fig. 2g) blocked the accumulation of macrophages in the brain after AC infection, decreased Chi3l3 (Fig. 2h), and relieved CNS pathology (Fig. 2i) at 22 dpi.

### Soluble antigens of AC larvae (sAg) play an expanding role in eosinophilic response in an airway allergy mouse model

**Fig. 3:**
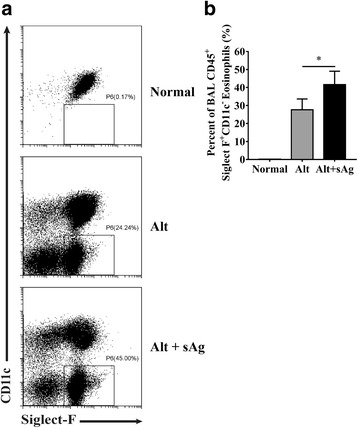
sAg aggravates eosinophilic response in an airway allergy C57BL/6 mouse model. **a** BAL eosinophil percentages were measured 4 days after daily administration of sAg 2 h before *Alternaria* exposure. **b** The results are representative or pooled from three repeat experiments, 3–5 mice per group. ^*^P < 0.05. Data information: In **b**, data are presented as mean ± SD. (Student’s *t* test)

A previous study showed that cellular infiltration around dead worms was more prominent than that around living ones [1], and these findings prompted us to investigate the function of sAg in eosinophil recruitment. *Alternaria* extract induces a pulmonary allergic reaction in mice, thus providing an ideal model for studies concerning the effect of helminths and their products on innate and adaptive immune responses [40, 41]. After 4 days of in vivo exposure to *Alternaria* and sAg, we observed an increase in eosinophils of bronchoalveolar lavage (BAL) fluid in C57BL/6 mice from the sAg administration group 2 h after *Alternaria* treatment (Fig. 3a, b).

These results demonstrate that the sAg stimulus may directly or indirectly affect the brain microenvironment during AC-induced eosinophilic meningitis.

### Network analysis and mathematical modeling predict a functional association between Chi3l3 and IL-13

**Fig. 4:**
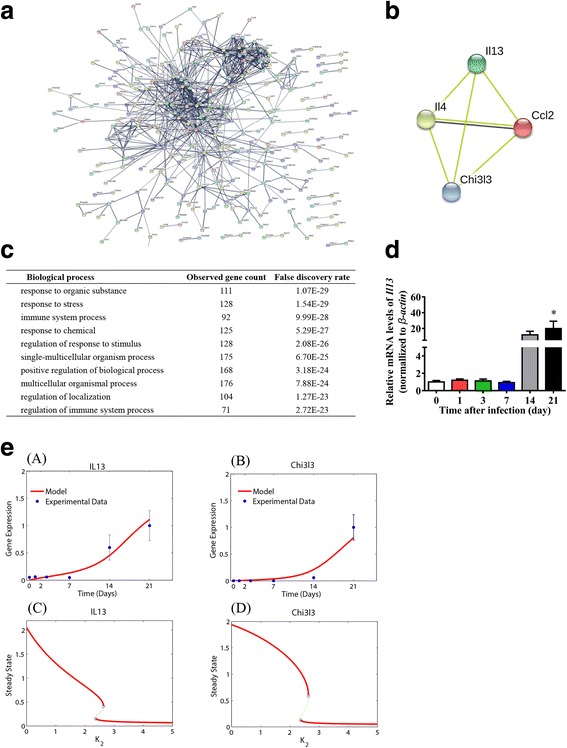
Functional protein association networks for the genes correlated with Chi3l3. **a** A full view of the protein association network for the genes highly correlated with Chi3l3. **b** The sub-network specifically centered on Chi3l3. **c** Functional enrichment for the genes in cluster 1 of gene expression patterns. The top 10 biological processes with the observed gene count as well as the false discovery rate are listed. **d** IL-13 mRNA levels in normal and AC-infected mouse brains. *P < 0.05, AC-infected 21 dpi group vs control group. **e** A mathematical model for Chi3l3-IL-13 positive feedback and bifurcation analysis. (A, B) Fitting the model to the experimental data. (C, D) Bistability of Chi3l3 and IL-13 with respect to the parameter value (*K*_2_). Data information: In (**d**–**e**), data are presented as mean ± SD. (Student’s *t* test)

To investigate the possible interactions of Chi3l3 with other genes, we constructed a functional interaction network of Chi3l3. We first calculated the Pearson correlation coefficients of Chi3l3 with all the other genes. The genes that were highly correlated with Chi3l3 (correlation coefficient greater than 0.8) were identified and input into the STRING database (https://string-db.org/). In this study, a medium confidence level (0.4) was selected to construct a functional gene association network (Fig. 4a). Then, a sub-network containing all genes interacting with Chi3l3 was extracted (Fig. 4b). IL-4, IL-13, and CCL2 were predicted to be functionally associated with Chi3l3.

The functional enrichment of genes in the Chi3l3 network was performed as shown in Fig. 4c. The significantly enriched biological processes include response to organic substance, response to stress, immune system process, response to chemical, regulation of response to stimulus, single-multicellular organism process, positive regulation of biological process, multicellular organismal process, regulation of localization, and regulation of immune system process.

Both RNA-seq data and qPCR measurements from in vivo experiments (Fig. 4d, e) indicated that Chi3l3 and IL-13 showed a high positive correlation and burst expression pattern. Therefore, we hypothesized that a positive feedback loop might exist between Chi3l3 and IL-13. To test this hypothesis, we developed an ordinary differential equation (ODE) model describing the positive feedback between Chi3l3 and IL-13 (Eqs. (2–3) in the “Methods” section] to quantitatively study the dynamics of Chi3l3 and IL-13 expression. We fitted the mode to the in vivo experimental data (Fig. 4d, e). A good agreement between the theoretical and experimental results (the mean squared error is 0.0849) supported the hypothesized positive feedback loop between Chi3l3 and IL-13. Moreover, bifurcation analysis of the model revealed a bistable switch of Chi3l3 and IL-13 with respect to the parameter *K*_2_ (Fig. 4f). As the value of *K*_2_ increased above ~ 2.63, the steady states of Chi3l3 and IL-13 then switched from high levels to low levels. The bistability of this system indicated the state transition of infection progression and heterogeneity of infection outcome between individuals. The switches induced by the increase in the value of *K*_2_ suggested that therapeutically inhibiting the IL-13 signaling pathway, including the IL-13 receptor, might ameliorate the AC infection.

### sAg aggravates the positive feedback loop between IL-13 and Chi3l3

**Fig. 5:**
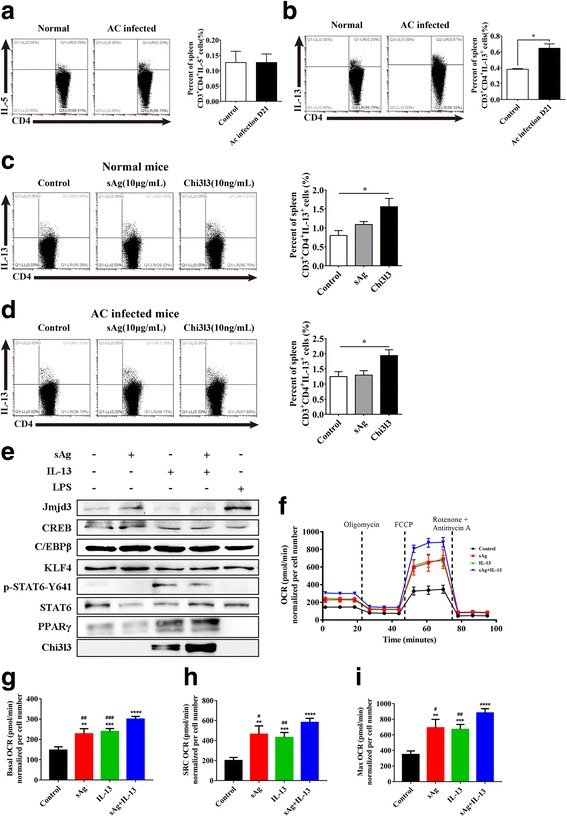
A positive feedback loop between Chi3l3 from macrophages and IL-13 from T lymphocytes in vitro*.* **a** The percent of IL-5^+^ cells in CD3^+^CD4^+^ spleen cells were detected by flow cytometry. **b** The percent of IL-13^+^ cells in CD3^+^CD4^+^ cells. *P < 0.05, AC-infected D21 (21 dpi) group vs control group. **c**, **d** Spleen cells isolated from normal mice and AC-infected mice were stimulated with sAg (25 μg/mL) or Chi3l3 (10 ng/mL) for 72 h in vitro. A summary of the percentages of IL-13^+^CD3^+^CD4^+^ cells following stimulation with sAg and Chi3l3 in normal mice. *P < 0.05, Chi3l3 group vs control group. **e** Western blot analysis of Chi3l3, JMJD3, CREB1, CEBPB, KLF4, Y641 phospho-STAT6, STAT6, and PPARγ of BMDMs in the presence of sAg, IL-13, sAg+IL-13, and LPS for 24 h. **f** Cells were cultured for 24 h in medium alone or treated with sAg, IL-13, or sAg+IL-13, and then, the OCR was monitored using the Seahorse Bioscience extracellular flux analyzer in real time. Dotted lines indicate incubation of cells with the indicated compounds. **g** Basal OCR of BMDMs cultured for 24 h in medium alone or treated with sAg, IL-13, or sAg+IL-13. **P < 0.01, ***P < 0.001, ****P < 0.0001, sAg group, IL-13 group, sAg+IL-13 group vs control group. ^#^*P* < 0.05, ^##^*P* < 0.01, ^###^*P* < 0.001, ^####^*P* < 0.0001, sAg group and IL-13 group vs sAg+IL-13 group. **h** SRC of BMDMs cultured for 24 h in medium alone or treated with sAg, IL-13, or sAg+IL-13. **P < 0.01, ***P < 0.001, ****P < 0.0001, sAg group, IL-13 group, sAg+IL-13 group vs control group. ^#^*P* < 0.05, ^##^*P* < 0.01, ^###^*P* < 0.001, ^####^*P* < 0.0001, sAg group and IL-13 group vs sAg+IL-13 group. **i** Maximal OCR of BMDMs cultured for 24 h in medium alone or treated with sAg, IL-13, or sAg+IL-13. **P < 0.01, ***P < 0.001, ****P < 0.0001, sAg group, IL-13 group, sAg+IL-13 group vs control group. ^#^*P* < 0.05, ^##^*P* < 0.01, ^###^*P* < 0.001, ^####^*P* < 0.0001, sAg group and IL-13 group vs sAg+IL-13 group. Data information: In (**a**–**d, f**–**h**), data are presented as mean ± SD (Student’s *t* test)

Flow cytometry analysis was carried out, which indicated IL-13 and IL-5 expression of CD3^+^CD4^+^ lymphocytes (Fig. 5a, b) in spleen, the most important peripheral immune organ. We observed a significant increase from 0.4 to 0.6% in IL-13^+^ expression of spleen-derived CD3^+^CD4^+^ cells, but not IL-5, which is consistent with the qPCR results of brains (Fig. 4e) (Additional file 2: Figure S2e).

To clarify whether Chi3l3 acts as a positive regulator of IL-13 cytokine production in our model, we cultured splenocytes of normal or AC-infected mice for 72 h in the presence of sAg or Chi3l3 followed by a 6-h PMA and ionomycin stimulation to increase cytokine production (Fig. 5c, d). We found that Chi3l3 enhanced spleen CD3^+^CD4^+^ T cell IL-13 secretion, with a percentage increase from 0.8 to 1.5% (Fig. 5c) and 0.8 to 1.9% (Fig. 5d) in normal mice and AC-infected mouse spleens, respectively.

Consistent with our previous study using RAW 264.7 cells [25], we found that BMDM Chi3l3 initiated by IL-13 was also promoted by sAg (Additional file 2: Figure S2a) (Fig. 5e). Thus, we hypothesized that uncontrolled Chi3l3 is achieved by an interlaced net of signaling pathways and transcription factors vital to the alternative activation of macrophages, including STAT6 phosphorylation [42], Histone H3 modification [43], PPARγ [44], C/EBPβ [45], and KLF4 [46] activation. We found that only BMDM PPARγ expression (Additional file 2: Figure S2b) (Fig. 5e) increased when stimulated with IL-13 and sAg plus IL-13, and sAg and IL-13 co-stimulation further enhanced Chi3l3. These data indicate that PPARγ is a strong activator of BMDM Chi3l3 expression in the sAg and IL-13 co-stimulation model. PPARγ is a known director of the oxidative phosphorylation process, which regulates M2 activation by controlling gene expression critical for metabolic reprogramming [44]. To gain insight into the metabolism of BMDMs in modulation of Chi3l3 expression, we measured the basal OCR, max OCR, and SRC OCR (spare respiratory capacity; the difference between basal OCR and max OCR) [31] level of BMDMs (Fig. 5g–i). Consistent with the increase in PPARγ, elevated OCR indicated that BMDMs displayed an enhanced oxidative phosphorylation metabolic phenotype (Fig. 5f).

Based on the results above, we verified the skewed cytokine pattern in brains of AC-infected mice, especially Chi3l3, which may be due to the positive feedback loop between IL-13 and Chi3l3 of inflammatory macrophages. This may be mediated by activating the PPARγ signaling pathway, which is associated with oxidative phosphorylation.

## Discussion

Previous studies have identified alternative activated macrophages as a hallmark of various parasitic diseases, including filarial nematode *Brugia malayi* [10], *Nippostrongylus brasiliensis* [11], *Trichinella spiralis* [12], *Schistosoma japonicum* [13], and *Heligmosomoides polygyrus* [14], along with high expression of Chi3l3 and major eosinophilic infiltration. The brain inflammation of AC-infected mice was characterized by major infiltration of eosinophils and “inflammatory” macrophages (Ly-6C^+^CCR2^+^CX3CR1^lo^) and a sharp increase in brain Chi3l3, an eosinophilic chemotactic factor also known as Chi3l3, ECF-L or Ym1. It is induced by allergens and helminths and was shown to exert chemotactic activity for eosinophils [47]. According to our previous work on AC-infected mice, Chi3l3 is one of the most highly expressed molecules in the brain, much greater than traditional chemotactic factors associated with eosinophils, such as Eotaxin, IL-5, and IL-13 [15, 17–19]. Moreover, it appears to be preferentially expressed in the brain compared to other organs, strongly indicating a close relationship between Chi3l3 and eosinophilic meningitis [35].

In this study, we first discovered an entirely different brain Chi3l3 expression pattern in permissive host rats compared to non-permissive host mice after AC infection. Interestingly, the dramatic increase of Chi3l3 was a unique feature of the non-permissive host mice. Chi3l3 appeared to be a major causative factor of the symptoms, as “inflammatory” macrophages were the major source of this molecule, and depletion of macrophages led to downregulation of Chi3l3 and relieved the brain symptoms of AC-infected mice. These findings are consistent with our previous results, which showed that Chi3l3 administration via the caudal vein resulted in a significant reduction in worm burden and augmented Chi3l3 protein levels in AC-infected mouse brains at 14 dpi compared with those of the saline administration AC-infected group [25]. Thus, continuous decomposition of worms is one reason for the rapid progression towards severity of this disease.

During the course of many helminth infections, M2 macrophages gradually accumulate in the lesion location, characterized by an increase of Chi3l3 [48], and both M2 macrophages and Chi3l3 may act as vital drivers of eosinophil recruitment in a Th2 cell-dependent manner [49], increasing severity of the lesions. Th2 cells are known to be critical sources of IL-4 and IL-13 [50], which are triggers of macrophage alternative activation.

Here, we selected IL-13 for further investigation of its function in AC infection and as a continuation of our previous study [25]. However, IL-4 and IL-13 share a signaling pathway (IL-4Rα/IL-13Rα1/STAT6) and similar functions [51]; thus, further research is needed to clarify the function of IL-4 in AC infection.

According to the mathematical analysis of our RNA-seq data, the dysregulated brain Chi3l3 in our AC-infected mice appeared to be strongly correlated with the IL-13 level. Through a series of in vitro experiments, we confirmed that sAg switched the active and metabolic status of macrophages to a M2 phenotype and substantially promoted Chi3l3 secretion via IL-13 in a PPAR-γ-dependent manner, suggesting that some helminth-derived molecules could be previously unknown sources of M2 activators. In turn, M2 macrophages enhanced Th2 polarization of spleen T lymphocytes through Chi3l3, forming a positive feedback loop, which may help explain the excess levels of Chi3l3 and IL-13 as well as the severe eosinophilic meningitis in the brain. As sAg aggravated the positive feedback loop between IL-13 and Chi3l3, breaking the loop between Chi3l3 and IL-13 is a promising treatment strategy for angiostrongyliasis.

Recent studies have shed light on the pharmacodynamic effects of helminth-derived molecules. *Trichinella spiralis* excretory/secretory (ES) antigens, as well as its recombinant protein r*Ts*P53, were reported to influence inflammatory cytokine production and the activation status of macrophages [12]. A *Brugia malayi* active cytokine mimic *Bm*-MIF-1 (macrophage migration inhibitory factor) was reported to induce Chi3l3 transcription in macrophages [52]. ES products from L3 larvae of *Nippostrongylus brasiliensis* can inhibit gene transcription of important inflammatory mediators in rat BAL cells. Filarial nematode-derived phosphorylcholine-containing glycoprotein ES-62, as well as its phosphorylcholine-based small molecule analogues, may have applications in the treatment of rheumatoid arthritis [53, 54], chronic asthma [55], lupus erythematosus [56], and atherosclerosis [57] in mouse models. *Trichuris suis*-derived glycan [58] and *Schistosoma japonicum*-derived sj16 [59] provided protection against ulcerative colitis and DSS-induced colitis, respectively. We hypothesized that further component analysis of the active ingredients of sAg might contribute to drug development and vaccine design for parasitic or autoimmune diseases.

## Conclusions

**Fig. 6:**
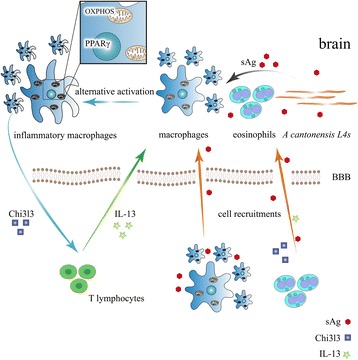
A summary of this article

We present evidences in favor of a key role for macrophage-derived Chi3l3 molecule in the infection of *Angiostrongylus cantonensis*, which aggravates eosinophilic meningitis induced by *Angiostrongylus cantonensis* via a IL-13-mediated positive feedback loop (Fig. 6). The results above indicate a great value for further research of angiostrongyliasis pathogenesis and implied that targeting chitinases and chitinase-like-proteins may be clinically beneficial in *Angiostrongylus cantonensis*-induced eosinophilic meningitis.

## Additional files

Additional file 1: Figure S1. The comparison between the expression levels of the top two genes in Table 1. Chi3l3 and LOC547349 were ranked according to the maximal changing rate of genes in cluster 1. Chi3l3 and LOC547349 have similar maximal changing rates, but Chi3l3 has a higher expression level. (TIFF 2791 kb)

Additional file 2: Figure S2. a–d. qPCR analysis of Chi3l3, PPARγ, JMJD3, and KLF4 of BMDMs in the presence of sAg, IL-13, sAg+IL-13, and LPS for 24 h. *E. IL*-5 mRNA levels in normal and AC-infected mouse brains. *P < 0.05, AC-infected 21 dpi group vs control group. (TIFF 739 kb)

## Abbreviations

2-DG: 2-Deoxyglucose
AC: *Angiostrongylus cantonensis*/*A. cantonensis*
BAL: Bronchoalveolar lavage
BMDM: Bone marrow-derived macrophage
CLPs: Chitinase-like proteins
CNS: Central nervous system
dpi.: Days post infection
ECAR: Extracelluar Acidification Rate
OCR: Oxygen Consumption Rate
sAg: Soluble antigens of *A. cantonensis* larvae (L4)
SRC: Spare respiratory capacity
TCGs: Significantly changing genes
