# Supplementary figures and images for: Chi3l3: a potential key orchestrator of eosinophil recruitment in meningitis induced by Angiostrongylus cantonensis
Source: J Neuroinflammation. 2018 Feb 2;15:31. doi: 10.1186/s12974-018-1071-2 (PMC5796390; doi:10.1186/s12974-018-1071-2)

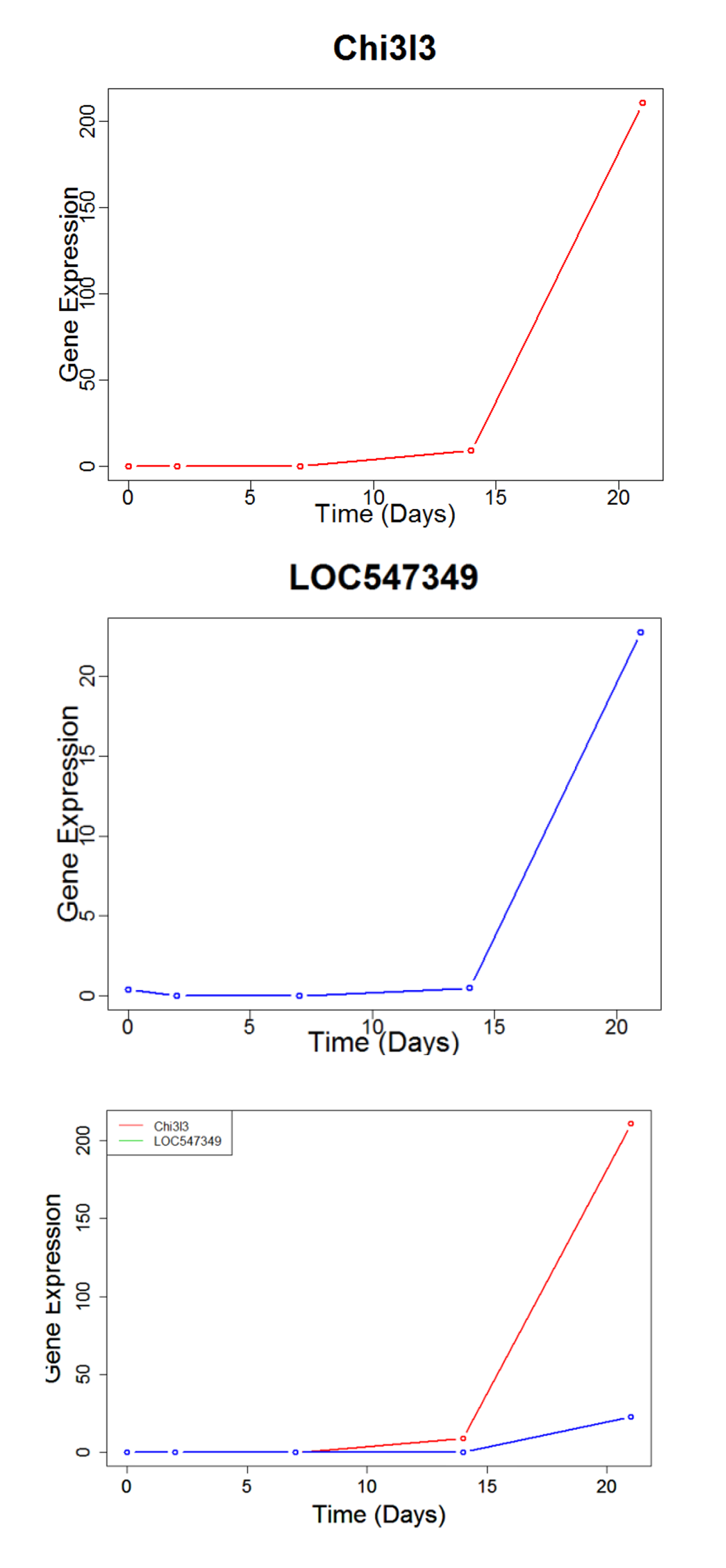

Supplement: Supplementary file 1 — The comparison between the expression levels of the top two genes in Table 1. Chi3l3 and LOC547349 were ranked according to the maximal changing rate of genes in cluster 1. Chi3l3 and LOC547349 have similar maximal changing rates, but Chi3l3 has a higher expression level. (TIFF 2791 kb) [file 12974_2018_1071_MOESM1_ESM.tif]

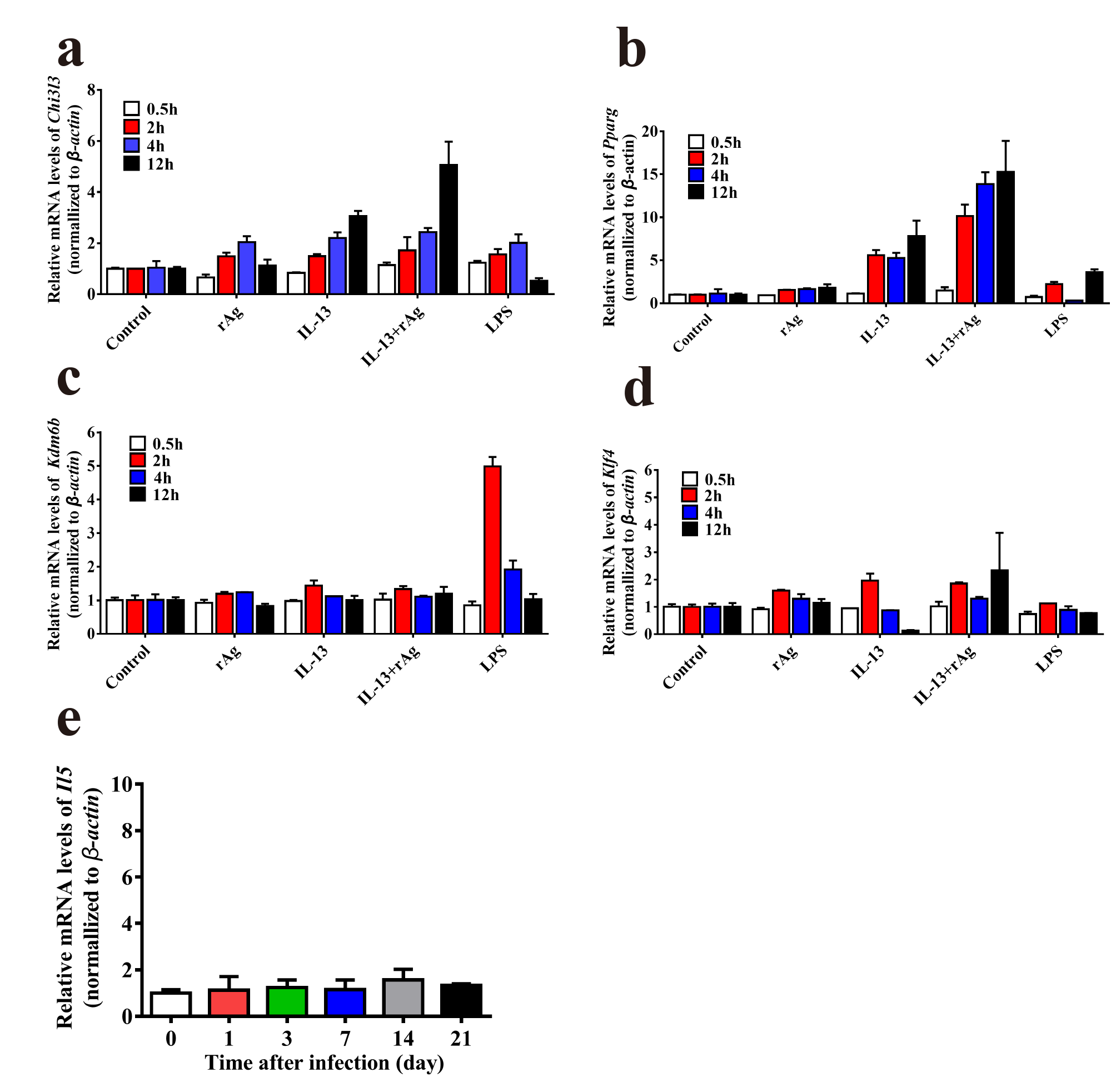

Supplement: Supplementary file 2 — a–d. qPCR analysis of Chi3l3, PPARγ, JMJD3, and KLF4 of BMDMs in the presence of sAg, IL-13, sAg+IL-13, and LPS for 24 h. E. IL-5 mRNA levels in normal and AC-infected mouse brains. *P < 0.05, AC-infected 21 dpi group vs control group. (TIFF 739 kb) [file 12974_2018_1071_MOESM2_ESM.tif]
